# Mental Health Challenges, Parenting Stress, and Features of the Couple Relationship in Parents of Children With Fragile X Syndrome

**DOI:** 10.3389/fpsyt.2022.857633

**Published:** 2022-04-01

**Authors:** Sarah Nelson Potter, Danielle J. Harvey, Audra Sterling, Leonard Abbeduto

**Affiliations:** ^1^MIND Institute, UC Davis Health, Sacramento, CA, United States; ^2^Department of Psychiatry and Behavioral Sciences, UC Davis Health, Sacramento, CA, United States; ^3^Department of Public Health Sciences, UC Davis Health, Sacramento, CA, United States; ^4^Waisman Center, University of Wisconsin-Madison, Madison, WI, United States; ^5^Department of Communication Sciences and Disorders, University of Wisconsin-Madison, Madison, WI, United States

**Keywords:** fragile X syndrome, mental health, parenting stress, couple relationships, parent-child relationships

## Abstract

**Background:**

Individuals with fragile X syndrome (FXS) have significant delays in cognition and language, as well as anxiety, symptoms of autism spectrum disorder, and challenging behaviors such as hyperactivity and aggression. Biological mothers of children with FXS, who are themselves *FMR1* premutation or full mutation carriers, are at elevated risk for mental health challenges in addition to experiencing stress associated with parenting a child with significant disabilities. However, little is known about fathers in these families, including the ways in which parental well-being influences the mother-father relationship and the impact of child characteristics on paternal and couple functioning.

**Method:**

The current study examined features of, and relationships between, parental well-being, couple well-being, and child functioning in 23 families of young boys with FXS. Mothers and fathers independently completed multiple questionnaires about their individual well-being, couple functioning, and child behavior. One parent per family also completed an interview about the child’s adaptive skills.

**Results:**

Results suggest that both mothers and fathers in these families experience clinically significant levels of mental health challenges and elevated rates of parenting stress relative to the general population. Findings also indicate that the couples’ relationship may be a source of strength that potentially buffers against some of the daily stressors faced by these families. Additionally, parents who reported less parenting stress had higher couples satisfaction and dyadic coping. Finally, parents of children with less severe challenging behaviors exhibited fewer mental health challenges, less parenting stress, and higher levels of both couples satisfaction and dyadic coping. Parents of children with higher levels of adaptive behavior also reported less parenting stress and higher couples satisfaction.

**Conclusion:**

Overall, this study provides evidence that families of children with FXS need access to services that not only target improvements in the child’s functioning, but also ameliorate parental stress. Family-based services that include both mothers and fathers would lead to better outcomes for all family members.

## Introduction

Fragile X syndrome (FXS) is an X-linked disorder that results from an expansion of a cytosine-guanine-guanine (CGG) sequence in the promoter region of the *FMR1* gene, located at Xq27.3, from the typical 35 or so repeats to greater than 200 repeats (1). Individuals with more than 200 CGG repeats have the full mutation (i.e., FXS), whereas individuals with 55 to 200 CGG repeats are premutation carriers. FXS is the leading inherited cause of intellectual disability (ID) (2), and children with FXS typically demonstrate delays in multiple domains of spoken language (3). In addition, these children also present with increased rates of challenging behaviors (e.g., aggression), symptoms of autism spectrum disorder (ASD), inattention, hyperarousal, and anxiety (4). Mothers of children with FXS are themselves either carriers of the *FMR1* premutation or the full mutation. These women experience a multitude of physical, mental health, and cognitive challenges (5, 6), which when compounded by the characteristics of the child with FXS, could negatively impact mother-child interactions and thus, child development. In addition, both mothers and fathers of children with FXS are likely to experience heightened levels of parenting stress due to the phenotypic characteristics associated with FXS in children (7), which may impede their ability to engage with their child in the sustained and productive interactions needed to facilitate the child’s development. Moreover, elevated parenting stress, which is likely given the challenging behaviors of individuals with FXS, is also likely to have a negative impact on the marital relationship for parents of children with FXS, which may further exacerbate the relationship between parents and their children (8). These relationships can be explained by the transactional model (9), which suggests that the development of a child results from the bidirectional effects between the child and the environment (e.g., interactions with a parent), such that experiences in the child’s environment are not considered independent of the child.

The majority of past studies on parenting in FXS have focused exclusively on the mother-child dyad. In doing so, these studies have neglected to consider the role that fathers play in child development or how features of the broader family environment may influence maternal or paternal behavior and child outcomes. The current study was designed to examine the broader family environment in families of young children with FXS, with a focus on maternal and paternal well-being, features of the mother’s and father’s relationship as a couple, and relationships between child characteristics and parent and couple well-being. A better understanding of parent and couple well-being in families of children with FXS, as well as the ways in which child characteristics influence these domains, will provide the foundation for developing interventions and services focused on improving outcomes for all family members.

### The Impact of *FMR1* Mutation Phenotypes on the Family System

The full mutation typically leads to hypermethylation and transcriptional silencing of the *FMR1* gene, causing a deficiency in, or absence of, the gene’s associated protein, FMRP (fragile X mental retardation 1 protein). FMRP is critical for early brain development, including synaptic protein synthesis and plasticity, as well as experience-dependent learning (4, 10, 11). In contrast, the premutation typically involves elevated levels of *FMR1* mRNA, which can lead to RNA toxicity. RNA toxicity is associated with reduced neuronal function, oxidative stress, chronic DNA damage repair changes, and ultimately the development of fragile X-associated tremor/ataxia syndrome (FXTAS) (5, 12) and other co-occurring physical and behavioral health challenges (described subsequently).

Because it is inherited, the presence of FXS in a family has far-reaching intergenerational effects, offering a unique opportunity to investigate the ways in which multiple family subsystems influence child outcomes. Nearly all males with FXS have ID (13), and many also experience a variety of other conditions, including hyperactivity, attention problems, anxiety, symptoms of ASD, aggressive and self-injurious behaviors, and abnormal sensory processing (14–17). Language is also significantly impaired in individuals with FXS, with some domains affected to an even greater extent than would be expected based upon their level of cognitive functioning (18–20). The combination of cognitive and psychiatric impairments in boys with FXS makes engaging in successful and productive interactions that are critical for the development of cognitive, language, and social skills more challenging (3).

The biological mothers of children with FXS are most often carriers of the *FMR1* premutation, although some also have the full mutation which causes FXS. Women with the full mutation are at an increased risk for experiencing mental health challenges, including anxiety and depression, as well as social deficits, including avoidance and withdrawal (5, 6, 12, 14, 21–23). Women with the *FMR1* premutation may also experience deficits in executive functioning, memory, and language (12, 24, 25). Moreover, cognitive functioning is variable in women with FXS, ranging from severe impairment to above average, with most of these women demonstrating IQs in the range of average to slightly below average (26). However, even some with average-range IQ scores can have a learning disability and/or deficits in executive functioning and attention (27). These cognitive phenotypic features of premutation and full mutation mothers are significant given that low maternal IQ is a risk factor for poorer child outcomes (28, 29).

Unfortunately, the mental health conditions that are experienced by the biological mothers of children with FXS can also be exacerbated by the stress they are likely to experience as a result of raising a child with significant challenges and impairments (30, 31). Furthermore, maternal depression and anxiety are associated with disrupted marital cohesion and decreased couples satisfaction (32, 33). Importantly, disruptions or problems in the marital relationship also affect paternal well-being, which may in turn negatively influence the quality of the relationship between father and child (34). Overall, poor parental and marital functioning have been repeatedly shown to contribute to negative child outcomes in neurotypical children (35–37).

Many previous studies have found that child characteristics, partner characteristics, and features of the marital relationship differentially affect mothers and fathers of children with disabilities, including ASD and Down syndrome (DS) (38–41). For example, in families including children with ASD, fathers are likely to be negatively affected by the child’s challenging behaviors to an even greater extent than are mothers (42). The same may be true in families affected by FXS given the symptom overlap between FXS and ASD (43). Moreover, maternal anxiety and depression—as well as the mother’s parenting stress—are also likely to take a significant toll on fathers in these families (44, 45), which could in turn negatively affect the father-child relationship. Maternal depressive symptoms, in particular, have been found to predict paternal well-being (i.e., depressive symptoms and pessimism) in families of children with ASD, DS, and FXS (45). Additionally, McCarthy et al. (7) found that both mothers and fathers of children with FXS reported high levels of stress, but that the predictors of stress differed between mothers and fathers with the strongest predictor of maternal stress being marital satisfaction and the strongest predictor of paternal stress being the child’s level of adaptive skills.

Very little else is known about fathers of children with FXS given that the majority of past studies have focused on the mother-child dyad. However, including both mothers and fathers in behavioral therapies and health care services positively contributes to a child’s success, especially for young children (46, 47). In order to maximize treatment gains for children with FXS and to improve well-being for the entire family system, researchers and clinicians need to develop a greater understanding of the challenges faced by families affected by FXS. This understanding will inform services and interventions for these families.

### Current Study

The current study was designed to examine multiple features of the family environment, including maternal and paternal mental health, stress associated with parenting, aspects of couple functioning, and relationships between child characteristics and these parental domains. We have four main aims.

Aim 1: Examine mental health challenges and parenting stress in biological mothers of children with FXS. We hypothesized that these mothers, compared to the general population, would report elevated levels of mental health challenges and parenting stress (5, 6, 48).

Aim 2: Examine mental health challenges and parenting stress in fathers of children with FXS and compare paternal and maternal mental health challenges and parenting stress. We hypothesized that fathers of children with FXS, compared to the general population, would report elevated levels of mental health challenges and parenting stress given the difficulties associated with parenting a child with significant challenges (7). We also hypothesized that fathers whose partners reported experiencing elevated levels of mental health challenges and parenting stress would themselves report elevated levels of mental health challenges and parenting stress compared to the general population based on past findings in families of children with ASD, DS, and FXS (45).

Aim 3: Examine relationships between aspects of the couple relationship (i.e., couples satisfaction and dyadic coping) and mothers’ and fathers’ mental health challenges and parenting stress. We hypothesized that couples satisfaction and dyadic coping would be negatively related to mental health challenges and parenting stress for both mothers and fathers (49).

Aim 4: Examine relationships between child characteristics (i.e., challenging behaviors, ASD symptoms, and adaptive behavior) and parental individual well-being (i.e., mental health challenges and parenting stress) and couple well-being (i.e., couples satisfaction and dyadic coping). We hypothesized that children with higher levels of behavior problems and ASD symptoms, and lower levels of adaptive behavior, would have parents who endorsed lower levels of individual well-being (30) and couple well-being (32) with fathers being affected by child characteristics to a greater extent than mothers (7).

## Materials and Methods

### Participants

The data for the current study were collected as part of a larger study investigating family relationships and parenting in families of children with FXS. Participants were 23 families of male children with FXS between the ages of 3;0 and 7;11 years, yielding a total of 69 participants including 23 fathers (22 biological fathers and one stepfather), 23 biological mothers, and 23 male children with FXS. Only families of male children were recruited because virtually all males with FXS have ID and language delays, whereas intellectual functioning and language abilities are more variable among females with FXS (3, 13). Eligibility criteria were (a) the child lived at home with both parents, (b) English was the primary language spoken in the home, and (c) the child had no uncorrected sensory or motor impairments that would limit his ability to participate in the study. Parents were asked to provide documentation of their child’s diagnosis of FXS as well as the mother’s *FMR1* premutation or full mutation status if available. Medical reports were required to confirm the child’s diagnosis of the *FMR1* full mutation, but verbal confirmation was accepted for the mother’s genetic status. The study was approved by the Institutional Review Board at the University of California, Davis in advance of recruitment, and both parents provided informed consent electronically *via* REDCap (Research Electronic Data Capture) (50, 51).

Participant characteristics are presented in Table 1. A majority of the participants identified as white and not Hispanic or Latinx. Twenty mothers were carriers of the *FMR1* premutation, two were carriers of the *FMR1* full mutation, and one had not been tested, so her genetic status was unknown. A majority of both mothers and fathers in the study had at least a bachelor’s degree and parent-reported household income indicated that most families were relatively well-resourced (i.e., approximately 50% had annual household incomes of $100,000 or above). All families resided in North America, with 13 United States states and two Canadian provinces represented. Data were collected between December 2019 and July 2021; therefore, the majority of families were tested during the COVID-19 pandemic. Only two families completed their participation in the study prior to the first community-diagnosed case in California on February 23, 2020.

**TABLE 1:** Family demographic characteristics.

*Individual characteristics* (*n* = 69)	Child		Mother		Father
**Age (years)**					
Mean (*SD*)	5.68 (1.45)		38.28 (6.00)		40.16 (5.86)
Range	3.07–7.90		25.15–50.43		27.79–51.46
**Race (*n*, %)**					
White	20 (87%)		21 (91%)		20 (87%)
Asian	2 (9%)		2 (9%)		3 (13%)
Mixed/Multiracial	1 (4%)		0 (0%)		0 (0%)
**Ethnicity (*n*, %)**					
Not Hispanic/Latinx	20 (87%)		20 (87%)		19 (83%)
Hispanic/Latinx	3 (13%)		3 (13%)		4 (17%)
***Parent characteristics* (*n* = 46)**		**Mother**		**Father**	
**Education (*n*, %)**					
Some high school		0 (0%)		1 (4%)	
High school/GED		1 (4%)		2 (9%)	
Some college/technical school		3 (13%)		2 (9%)	
Associate’s/technical degree		2 (9%)		2 (9%)	
Bachelor’s degree		8 (35%)		9 (39%)	
Master’s/other advanced degree		9 (39%)		7 (30%)	
**Employment (*n*, %)**					
Not currently employed		9 (39%)		4 (17%)	
Part-time		7 (30%)		0 (0%)	
Full-time		7 (30%)		19 (83%)	
**Previous or current psychiatric diagnosis^1^**		7 (30%)		3 (13%)	
***Family characteristics* (*n* = 23)**					
**Annual household income (*n*, %)**					
Under $50,000			1 (4%)		
50,001–$100,000			8 (35%)		
100,001–$150,000			5 (22%)		
150,001–$250,000			7 (30%)		
Unknown			2 (9%)		
**Additional siblings in family (*n*, %)^2^**					
0			3 (13%)		
1			13 (56%) [4]		
2			5 (22%) [4]		
3			2 (9%)		

*The individual percentage values are rounded and may not total 100%. ^1^According to parent report. ^2^Number of families in which a sibling also had a disability noted in brackets.*

### Measures

In order to address the aims stated above, mothers and fathers independently completed multiple questionnaires *via* REDCap (50, 51) and one parent completed an interview about the child. REDCap is a secure, web-based software platform designed to support data capture for research studies, providing (1) an intuitive interface for validated data capture; (2) audit trails for tracking data manipulation and export procedures; (3) automated export procedures for seamless data downloads to common statistical packages; and (4) procedures for data integration and interoperability with external sources.

Parents completed questionnaires pertaining to: (a) their individual well-being, including the Symptom Checklist-90-Revised (SCL-90-R) (52) and the Parenting Stress Index—Fourth edition, Short Form (PSI-4-SF) (53); (b) couple functioning, including the Couples Satisfaction Index (CSI-32) (54) and the Dyadic Coping Inventory (DCI) (55, 56); and (c) child functioning and behavior, including the Aberrant Behavior Checklist, 2nd edition (ABC-2) (57) and the Social Responsiveness Scale, 2nd edition (SRS-2) (58). One parent also completed the Vineland Adaptive Behavior Scales, 3rd edition (Vineland-3) (59) as an interview to assess the child’s adaptive behavior.

### Individual-Level Parent Measures

#### Symptom Checklist-90-Revised

The SCL-90-R (52) is a 90-item scale that measures mental health symptoms along the following dimensions: Somatization, Obsessive-Compulsive, Interpersonal Sensitivity, Depression, Anxiety, Hostility, Phobic Anxiety, Paranoid Ideation, Psychoticism, and additional symptoms, yielding a Positive Symptom Total, a Positive Symptom Distress Index, and a Global Severity Index. Lower scores indicate lower levels of mental health challenges. T-scores from each dimension and the Global Severity Index (representing overall mental health challenges) were reported and used in analyses. Overall, a T-score of 63 or above (equivalent to the 90th percentile) on the Global Severity Index, or two or more scores of 63 or above on any dimension, suggest clinically significant levels of mental health challenges. The scale takes approximately 15 min to complete.

#### Parenting Stress Index—Fourth Edition, Short Form

The PSI-4-SF (53) is a 36-item scale that measures parenting stress in the domains of anxiety, mood, relationships, attachment, and family mental health and functioning. Like the 120-item PSI, the short form provides scores on the following subscales: (1) Parental Distress, (2) Parent-Child Dysfunctional Interaction, and (3) Difficult Child. The three domains include items related to: an individual’s adjustment to parenting (Parental Distress), the relationship between the parent and child (Parent-Child Dysfunctional Interaction), and a parent’s perception of the child’s behavior (Difficult Child). Lower scores on these subscales indicate lower levels of parenting stress. T-scores and percentiles from these dimensions as well as the Total Stress score were reported and used in analyses. This questionnaire takes approximately 10 min to complete.

### Couple-Level Parent Measures

#### Couples Satisfaction Index

The CSI-32 (54) is a 32-item scale that measures satisfaction in the couple’s relationship, with higher total scores indicating higher satisfaction. Item-response theory was used to develop the CSI-32 from a set of 180 relationship satisfaction items administered to over 5,000 individuals. Example items include, “In general, how often do you think that things between you and your partner are going well?” and “How good is your relationship compared to most?”. Compared to previous measures of relationship satisfaction, the CSI-32 demonstrates higher precision and has strong internal consistency and construct validity. Scores on the CSI-32 range from 0 to 161. Scores below 104.5 indicate notable relationship dissatisfaction. Total scores were used in analyses. This scale takes approximately 10 min to complete.

#### Dyadic Coping Inventory

The DCI (55, 56) is a 37-item scale that measures perceived communication and coping that occurs in relationships when one or both partners are experiencing stress. In this measure, dyadic coping is assessed as a multidimensional construct that includes the following components: supportive, delegated, negative, and joint (common) coping. The DCI helps to assess an individual’s perceptions about both the quality and quantity of the partner’s support in the dyadic relationship. Example items include, “My partner shows empathy and understanding to me,” and “I listen to my partner and give him/her space and time to communicate what really bothers him/her.” Scores on the DCI range from 35 to 175. Scores below 111 indicate below average dyadic coping, whereas scores above 145 indicate above average coping. Total scores from this measure were used in analyses. This scale takes approximately 10 min to complete.

### Child Measures

#### Aberrant Behavior Checklist, 2nd Edition

The ABC-2 (57) is a 58-item scale developed to assess challenging behaviors of individuals with developmental disabilities in several domains. For this study, subscale scoring based on the revised FXS-specific factor structure from Sansone et al. was used (60). The following factors are included in the FXS-specific subscale scoring: Irritability, Socially Unresponsive/Lethargic, Stereotypy, Hyperactivity, Inappropriate Speech, and Social Avoidance. Total raw scores for the FXS-specific factor structure scoring range from 0 to 165 and were used in the present analyses. This checklist takes approximately 10 min to complete.

#### Social Responsiveness Scale, 2nd Edition

The SRS-2 (58) is a 65-item scale used to assess social impairments commonly associated with ASD. Mothers and fathers independently completed either the Preschool (2½–4½ years) or School-Aged (4–18 years) form depending on their child’s chronological age. On the SRS-2, the following subscales are included in addition to a total score: Social Awareness, Social Cognition, Social Communication, Social Motivation, and Restricted Interests and Repetitive Behavior. DSM-5 compatible subscale scores include a Social Communication and Interaction (SCI) score and a Restricted Interests and Repetitive Behavior (RRB) score. SCI, RRB, and Total T-scores were used in analyses. The scale takes approximately 15 min to complete.

#### Vineland Adaptive Behavior Scales, 3rd Edition

The Vineland-3 (59) measures adaptive behavior across the following domains: Communication, Socialization, Daily Living Skills, Motor Skills, and Maladaptive Behavior. For this study, only the Communication, Socialization, and Daily Living Skills domains were administered. The Vineland-3 was administered as an interview by a trained examiner using Q-Global, a web-based platform for online administration. The child’s primary caregiver (as reported by the parents) was interviewed over the phone or *via* a secure teleconferencing platform (i.e., Skype for Business or Zoom). The Vineland-3 is a norm-based instrument with a mean standard score of 100 and a standard deviation of 15. The Adaptive Behavior Composite score as well as the Communication, Daily Living Skills, and Socialization domain standard scores were used in analyses. The Vineland-3 interview takes approximately 1 to 2 h to complete.

The SCL-90-R, PSI-4-SF, CSI-32, DCI, ABC-2, and SRS-2 are traditionally paper-and-pencil measures. They were modified so that they could be completed in packages (i.e., individual parent measures, couple measures, child measures) as online surveys *via* REDCap during different days of the study.

### Analysis Plan

Analyses were conducted using Stata 14.2. All variables were visually inspected to check for model assumptions of normality and homoscedasticity of the residuals. Tests for skewness and kurtosis were also examined. Transformations and nonparametric alternatives were considered for any data that did not meet parametric assumptions.

To address the first and second aims, descriptive summaries of mothers’ and fathers’ mental health challenges and parenting stress (the outcomes variables) were reported and compared to levels reported in the general population and to each other. Then, interspousal correlations were calculated to determine the degree of correspondence between mothers’ and fathers’ ratings of mental health challenges and parenting stress. Comparisons of mothers’ and fathers’ mean scores on the SCL-90-R and PSI-4-SF were also conducted.

To address the third aim examining aspects of the couple relationship and parental mental health challenges and parenting stress, descriptive summaries of the outcome variables (i.e., couples satisfaction and dyadic coping) were reported and mean scores for mothers and fathers were compared. Interspousal correlations were then calculated to determine the degree of correspondence between mothers’ and fathers’ ratings of couples satisfaction and dyadic coping. Comparisons of mothers’ and fathers’ mean scores on the CSI-32 and DCI were also reported. To address the fourth aim examining relationships between child characteristics and parental and couple functioning, descriptive summaries of the predictor variables (i.e., challenging behaviors, ASD symptoms, and adaptive behavior) and interspousal correlations were reported. Comparisons of mothers’ and fathers’ mean scores on the ABC-2 and SRS-2 were also conducted.

Additionally, given that data collected from couples are considered to be non-independent observations (61), a multilevel modeling (MLM) approach, also known as hierarchical linear modeling (HLM), was used for Aims 3 and 4 (62). In this approach, the data from each partner is nested within a group that has an *N* of 2 (63). Effect coding was used for parent sex such that Male = 1 and Female = −1. Continuous predictors were centered to their respective grand means.

Visual inspection of the variables and tests for skewness and kurtosis indicated that the CSI-32 scores for mothers and fathers were negatively skewed. A cubic transformation of the variable reduced the negative skewness and was used to examine correlations between the CSI-32 and the other measures. To avoid difficulty in interpreting the cubic transformation of the CSI-32 variable in a multilevel model, a new categorical variable was created that reduced the significant negative skewness of the CSI-32 scores (confirmed using the Shapiro-Wilk test of normality). For this new variable, ranges of the CSI-32 score were given a value of 1–8 (e.g., scores ≤49 had a value of 1, scores from 50 to 99 had a value of 2, scores from 100 to 109 had a value of 3).

Intraclass correlation coefficients (ICCs) were then calculated to estimate the proportion of the total variation in the dependent variables that exists between versus within couples for Aims 3 and 4. The dependent variables for Aim 3 included couples satisfaction and dyadic coping (total raw scores from the CSI-32 and DCI, respectively). The dependent variables for Aim 4 included couples satisfaction and dyadic coping, as well as mental health challenges (SCL-90-R GSI T-score) and parenting stress (PSI-4-SF Total Stress T-score). Next, multilevel models were specified to examine the outcomes for Aims 3 and 4. For Aim 3, separate models for couples satisfaction and dyadic coping were conducted. The strong and significant association between the variables for parenting stress and mental health challenges did not allow for them both to be included in the models for Aim 3; the parenting stress measure was chosen as it was more strongly associated with both couples satisfaction and dyadic coping than the measure of mental health challenges for both mothers and fathers.

As an example, the model for couples satisfaction (*CS*) was specified as follows, with parenting stress (*PS*) and parent sex (*sex*) set as predictors at Level 1. Covariates included parent age (*age*) and parent education (*edu*). Parenting stress, parent age, and parent education were continuous predictors. In this example, random effects were not included at Level 2 for parenting stress, parent sex, parent age, or parent education; therefore, the effects of these predictors on the outcome (*CS*) are fixed. However, a family level random effect for the intercept was included at Level 2:

Level 1:


$$C\,S_{i\,j}=\,\beta_{0\,j}+\beta_{1\,j}\,\left(P\,S_{i\,j}\right)+\beta_{2\,j}\,\left(s\,e\,x_{i\,j}\right)+\beta_{3\,j}\,\left(a\,g\,e_{i\,j}\right)+\beta_{4\,j}\,\left(e\,d\,u_{i\,j}\right)+e_{i\,j}$$

Level 2:


$$\beta_{0\,j}=\gamma_{00}+\mu_{0\,j}$$

$$\beta_{1\,j}=\gamma_{10}$$

$$\beta_{2\,j}=\gamma_{20}$$

$$\beta_{3\,j}=\gamma_{30}$$

$$\beta_{4\,j}=\gamma_{40}$$

Composite:


$$C\,S_{i}\,j=\left[\gamma_{00}+\gamma_{10}\,\left(P\,S_{i\,j}\right)+\gamma_{20}\,\left(s\,e\,x_{i\,j}\right)+\gamma_{30}\,\left(a\,g\,e_{i\,j}\right)+\gamma_{40}\,\left(e\,d\,u_{i\,j}\right)\right]+\left[\mu_{0\,j}+e_{i\,j}\right]$$

For Aim 4, separate models were specified for mental health challenges, parenting stress, couples satisfaction, and dyadic coping using the same model building strategy employed in the Aim 3 models. Interactions between parent sex and child variables were included to examine the differential effects of child characteristics on mothers and fathers. Child variables were entered into the models as continuous predictors.

## Results

### Aims 1 and 2

Aim 1 examined mental health challenges and parenting stress in biological mothers of children with FXS. Aim 2 examined mental health challenges and parenting stress in fathers of children with FXS and compared maternal and paternal mental health challenges and parenting stress. Table 2 displays descriptive statistics for the SCL-90-R dimension scores, the measure of mental health challenges, for both mothers and fathers. Paired samples *t*-tests and Wilcoxon signed-ranks tests (when appropriate) confirmed that there were no statistically significant differences between mothers’ and fathers’ standardized scores on the SCL-90-R (all *p*-values ≥ 0.277).

**TABLE 2:** Mother-father comparisons of SCL-90-R dimension scores (*n* = 46).

	Mean T-score (*SD*) Range		*n* (%) with T-score ≥ 63		Interspousal correlations
SCL-90-R Dimension	Mothers	Fathers	Mothers	Fathers	ρ (*p*-value)
Somatization	50.70 (9.24) 35–70	53.57 (12.43) 37–77	3 (13%)	4 (17%)	−0.22 (0.320)
Obsessive-Compulsive	58.52 (10.57) 37–78	59.39 (12.23) 39–80	9 (39%)	9 (39%)	0.14 (0.516)
Interpersonal Sensitivity	57.35 (11.82) 39–80	58.04 (13.61) 41–80	10 (43%)	9 (39%)	0.03 (0.892)
Depression	57.57 (11.15) 34–75	59.22 (13.17) 38–80	8 (35%)	9 (39%)	−0.03 (0.877)
Anxiety	52.52 (10.92) 37–73	51.65 (11.94) 40–73	4 (17%)	5 (22%)	0.11 (0.626)
Hostility	57.30 (8.74) 40–74	56.35 (12.46) 41–80	6 (26%)	7 (30%)	−0.15 (0.485)
Phobic Anxiety	52.44 (10.76) 44–77	52.13 (9.12) 47–71	4 (17%)	5 (22%)	0.29 (0.186)
Paranoid Ideation	51.57 (10.48) 41–72	55.22 (14.61) 41–80	3 (13%)	7 (30%)	0.25 (0.245)
Psychoticism	52.83 (10.56) 44–80	51.74 (11.95) 44–80	6 (26%)	5 (22%)	0.22 (0.314)
Global Severity Index	56.52 (11.09) 30–79	56.78 (14.24) 34–80	6 (26%)	8 (35%)	0.02 (0.912)

Table 2 also displays information regarding the number of parents who met the instrument’s cutoff for clinical significance on the SCL-90-R dimensions (i.e., a T-score of 63 or above on the Global Severity Index, or two or more scores of 63 or above on any dimension). According to these criteria, 10 out of 23 (43%) mothers and 10 out of 23 (43%) fathers reported clinically significant levels of mental health challenges. In six families, both the mother and father met the clinical criteria. The rates of clinically significant mental health challenges in the present sample are higher than what is reported in the general population for both males and females. Specifically, recent estimates suggest that approximately 24.5% of women in the United States suffer from any mental illness compared to approximately 16.3% of men; for these estimates, which were reported in the 2019 National Survey on Drug Use and Health conducted by the United States Department of Health and Human Services, adults aged 18 or older were classified as having any mental illness if they met DSM-IV criteria for any mental, behavioral, or emotional disorder in the past year (excluding any developmental or substance use disorders) (64).

Table 3 displays descriptive statistics for the PSI-4-SF domain scores, the measure of parenting stress, for both mothers and fathers. Much like the SCL-90-R, paired samples *t*-tests and Wilcoxon signed-ranks tests (when appropriate) confirmed that there were no statistically significant differences between mothers’ and fathers’ standardized scores on the PSI-4-SF (all *p*-values ≥ 0.275). On the PSI-4-SF, scores that fall between the 16th and 84th percentiles are considered within the normal range, scores between the 85th and 89th percentiles are considered high, and scores at the 90th percentile and above are within the clinically significant range. Table 4 shows the number of mothers and fathers who reported scores within each of these ranges on the PSI-4-SF domains. Notably, a majority of mothers and fathers reported normal levels of parenting stress on the Parental Distress and Parent-Child Dysfunctional Interaction domains. However, a fairly large proportion of both mothers (43%) and fathers (30%) reported clinically significant levels of parenting stress in the Difficult Child domain.

**TABLE 3:** Mother-father comparisons of PSI-4-SF domain scores (*n* = 46).

	Mothers		Fathers		Interspousal Correlation
	Mean (*SD*) Range		Mean (*SD*) Range		
PSI-4-SF Domain	T-Score	Percentile	T-Score	Percentile	ρ (*p*-value)
Parental Distress	54.09 (12.41) 34–79	61.39 (30.57) 3–99	50.74 (10.30) 34–72	54.30 (28.22) 3–99	0.21 (0.348)
P-C Dysfunctional Interaction	55.30 (8.55) 41–76	68.78 (19.97) 24–99	55.35 (9.19) 40–81	68.39 (18.55) 19–99	0.04 (0.849)
Difficult Child	59.91 (9.34) 42–80	78.09 (19.51) 26–99	57.70 (10.56) 35–77	72.26 (24.89) 6–99	0.30 (0.170)
Total Stress	57.04 (10.24) 41–81	69.96 (23.21) 19–99	54.87 (9.73) 35–79	66.09 (23.28) 4–99	0.23 (0.288)

**TABLE 4:** PSI-4-SF domain percentile scores (*n* = 46).

	Percentile Range *n* (%)					
	Normal (16–84)		High (85–89)		Clinically Significant (90+)	
PSI-4-SF Domain	Mothers	Fathers	Mothers	Fathers	Mothers	Fathers
Parental Distress	17 (74%)	21 (91%)	1 (4%)	0 (0%)	5 (22%)	2 (9%)
P-C Dysfunctional Interaction	18 (78%)	20 (87%)	1 (4%)	1 (4%)	4 (17%)	2 (9%)
Difficult Child	12 (52%)	15 (65%)	1 (4%)	1 (4%)	10 (43%)	7 (30%)
Total Stress	17 (74%)	18 (78%)	0 (0%)	2 (9%)	6 (26%)	3 (13%)

*The individual percentage values are rounded and may not total 100%. Parents whose percentile scores were below 16 were included in the Normal category.*

To determine the degree of correspondence between mothers’ and fathers’ ratings of mental health challenges and parenting stress, interspousal correlations were calculated. Tables 2, 3 also display interspousal correlations for the SCL-90-R dimensions and PSI-4-SF domains, respectively. Given that some of the scores for these two measures were not normally distributed, Spearman’s rank-order correlations were used. Unexpectedly, no significant correlations were found between mothers’ and fathers’ scores across these measures, despite the overlap within families in clinically significant cases on the SCL-90-R and the fact that there were no significant differences in the mean scores between mothers and fathers on either of the measures.

### Aim 3

Aim 3 examined relationships between aspects of the couple relationship (i.e., couples satisfaction and dyadic coping) and mothers’ and fathers’ mental health challenges and parenting stress. The ICC for couples satisfaction indicated that 76.6% of the variation was due to between-couples factors whereas 23.4% was due to within-couple factors. For dyadic coping, 42.1% of the variation was due to between-couples factors whereas 57.9% was due to within-couple factors. Table 5 displays descriptive statistics for mothers’ and fathers’ CSI and DCI scores as well as interspousal correlations, which indicate the degree of correspondence between their ratings of couples satisfaction and dyadic coping.

**TABLE 5:** Mother-father comparisons of couple relationship measures (*n* = 46).

	Mean (*SD*) Range		*r* (*p*-value)
Variable	Mothers	Fathers	Interspousal Correlation
Couples Satisfaction^1^	123.74 (30.28) 33–155	124.57 (30.82) 43–158	0.76*** (<0.001)
Dyadic Coping	130.78 (18.74) 98–167	125.83 (16.26) 100–156	0.44* (0.038)

*^1^CSI-32 raw score underwent cubic transformation to reduce negative skewness.*

^∼^p < 0.100, *p < 0.050, **p < 0.010, ***p < 0.001. The bold values indicates significant values.

Unlike the measures of mental health challenges and parenting stress, interspousal correlations indicated that there were significant correspondences between mothers’ and fathers’ scores on both the CSI-32 and DCI, with mean scores indicating average levels of couples satisfaction and dyadic coping for both mothers and fathers. Additionally, paired samples *t*-tests confirmed that there were no statistically significant differences between mothers’ and fathers’ scores on the CSI-32 [*t*(22) = −0.30, *p* = 0.766] or the DCI [*t*(22) = 1.27, *p* = 0.217]. Overall, only six mothers (26%) and four fathers (17%) reported notable relationship dissatisfaction on the CSI-32. Additionally, on the DCI, five mothers and five fathers (22%) reported below average levels of dyadic coping, 13 mothers (57%) and 14 fathers (61%) reported average levels of dyadic coping, and five mothers (22%) and four fathers (17%) reported above average levels of dyadic coping.

#### Prediction of Couples Satisfaction

Table 6 presents the results of the MLM analysis for couples satisfaction. As expected, there was a significant main effect of parenting stress on couples satisfaction (*p* = 0.010), such that higher levels of parenting stress predicted reduced couples satisfaction. There were no significant main effects of parent sex, parent age, or parent education on couples satisfaction.

**TABLE 6:** Multilevel model results for Aim 3 (*n* = 46).

	β (SE)	
	Couples satisfaction	Dyadic coping
**Fixed Effects**		
Intercept	5.30*** (0.36)	128.30*** (2.32)
Parenting Stress	−0.07* (0.03)	−0.94*** (0.23)
Parent Sex	0.01 (0.20)	−2.16 (1.99)
Parent Age	−0.10 (0.06)	−0.71^∼^ (0.41)
Parent Education	−0.21 (0.19)	−3.47* (1.61)
**Random Effects**		
Residual ($\sigma_{e}^{2}$)	1.72	169.75
Intercept ($\sigma_{u\,0}^{2}$)	2.10	39.30
**Goodness-of-fit**		
AIC	205.33	376.16
BIC	218.13	388.96

^∼^p < 0.100, *p < 0.050, **p < 0.010, ***p < 0.001. The bold values indicates significant values.

#### Prediction of Dyadic Coping

Table 6 also presents the results of the MLM analysis for dyadic coping. As predicted, there was a significant main effect of parenting stress on dyadic coping (*p* < 0.001), such that higher levels of parenting stress predicted poorer dyadic coping. There was also a significant main effect of parent education on dyadic coping (*p* = 0.031), such that higher levels of education predicted poorer dyadic coping. There was no significant main effect of parent sex on dyadic coping, but there was a marginally significant main effect of parent age on dyadic coping (*p* = 0.083), such that older age predicted poorer dyadic coping.

### Aim 4

Aim 4 examined the contributions of child challenging behaviors, ASD symptoms, and adaptive behavior to parental individual well-being (i.e., mental health challenges and parenting stress) and couple well-being (i.e., couples satisfaction and dyadic coping). The ICC for mental health challenges indicated that 3.1% of the variation was due to between-couples factors whereas 96.9% was due to within-couple factors. For parenting stress, 27.0% of the variation was due to between-couples factors whereas 73.0% was due to within-couple factors. Visual inspection of the variables and tests for kurtosis and skewness indicated that several of the ABC-2 subscale scores and one of the SRS-2 subscale scores were not normally distributed. Tables 7, 8 display descriptive statistics for parents’ ABC-2 and SRS-2 scores, respectively, as well as interspousal correlations, which indicate the degree of correspondence between their ratings of child challenging behaviors and ASD symptoms on these measures. Given that some of the scores for these two measures were not normally distributed, Spearman’s rank-order correlations were used.

**TABLE 7:** Mother-father comparisons of ABC-2 subscale raw scores (*n* = 46).

	Mean (*SD*) range		ρ (*p*-value)
ABC-2 Subscale	Mothers	Fathers	Interspousal correlation
Irritability	18.26 (10.62) 1–38	17.44 (11.54) 1–47	0.36^∼^ (0.096)
Socially Unresponsive/Lethargic	4.52 (5.86) 0–23	5.17 (4.65) 0–20	0.23 (0.299)
Stereotypy	6.09 (2.78) 1–12	4.96 (3.76) 0–13	0.24 (0.268)
Hyperactivity	14.04 (8.07) 2–27	11.00 (5.42) 1–24	0.54** (0.007)
Inappropriate Speech	4.48 (3.85) 0–11	3.17 (2.29) 0–8	0.48* (0.021)
Social Avoidance	1.39 (2.61) 0–9	1.52 (2.39) 0–9	0.33 (0.123)
Total Score	49.39 (27.71) 12–98	43.87 (24.70) 10–103	0.36^∼^ (0.094)

^∼^p < 0.100, *p < 0.050, **p < 0.010, ***p < 0.001. The bold values indicates significant values.

**TABLE 8:** Mother-father comparisons of SRS-2 subscale T-scores (*n* = 46).

	Mean (*SD*) range		ρ (*p*-value)
SRS-2 Subscale	Mothers	Fathers	Interspousal correlation
Social Communication and Interaction	69.00 (11.00) 49–90	66.83 (9.53) 49–85	0.56** (0.005)
Restricted Interests and Repetitive Behavior	75.48 (11.63) 57–92	69.17 (10.35) 54–90	0.41* (0.049)
Total Score	70.87 (10.96) 51–91	67.57 (9.40) 51–87	0.52* (0.011)

^∼^p < 0.100, *p < 0.050, **p < 0.010, ***p < 0.001. The bold values indicates significant values.

Interspousal correlations indicated significant correspondences between mothers’ and fathers’ scores on the ABC-2 for the Hyperactivity and Inappropriate Speech subscales on the ABC-2, but not for the other four subscales. On the SRS-2, there were significant correspondences between mothers’ and fathers’ scores on the SCI and RRB subscale T-scores as well as the Total T-score. Additionally, paired samples *t*-tests and Wilcoxon signed-ranks tests (when appropriate) confirmed that there were no significant differences between mothers’ and fathers’ subscale and total scores on these measures except for the ABC-2 Hyperactivity subscale, *t*(22) = 2.11, *p* = 0.046, and the SRS-2 RRB subscale, *Z* = 2.27, *p* = 0.023. For both of these subscales, mothers endorsed higher scores than fathers. Furthermore, according to the SRS-2 Total Score guidelines, scores can be classified as within normal limits (T-scores ≤ 59), in the mild range (T-scores = 60 to 65), in the moderate range (T-scores = 66 to 75), or in the severe range (T-scores ≥ 76). Table 9 displays the number of mothers and fathers who reported scores within each of these ranges on the SRS-2. Table 10 displays descriptive statistics for the Vineland-3 scores, and Tables 11, 12 display correlations between the measures of couple (i.e., CSI-32 and DCI total raw scores), individual (i.e., SCL-90-R Global Severity Index and PSI-4-SF Total Stress T-scores), and child (ABC-2 total raw score and SRS-2 Total T-Score) functioning for mothers and fathers, respectively.

**TABLE 9:** Mother-father comparison of SRS-2 total score severity range (*n* = 46).

	*n* (%)	
Range	Mothers	Fathers
Within normal limits	3 (13%)	5 (22%)
Mild	4 (17%)	4 (17%)
Moderate	9 (39%)	8 (35%)
Severe	7 (30%)	6 (26%)

*The individual percentage values are rounded and may not total 100%.*

**TABLE 10:** Vineland-3 scores (*n* = 23).

Vineland-3 Domain	Mean (*SD*)	Range
Communication	62.65 (18.31)	20–96
Daily Living Skills	69.13 (10.09)	52–93
Socialization	72.52 (14.51)	40–104
Adaptive Behavior Composite	67.70 (11.87)	39–89

**TABLE 11:** Pearson correlations for maternal variables (*n* = 23).

Variable	1.	2.	3.	4.	5.	6.
1. SCL-90-R^1^	1.00					
2. PSI-4-SF^2^	0.73*** (<0.001)	1.00				
3. CSI-32^3^	−0.29 (0.181)	−0.44* (0.037)	1.00			
4. DCI^4^	−0.27 (0.212)	−0.53* (0.010)	0.81*** (<0.001)	1.00		
5. ABC-2^5^	0.65** (0.001)	0.64** (0.001)	−0.55** (0.006)	−0.54** (0.007)	1.00	
6. SRS-2^6^	0.29 (0.188)	0.32 (0.139)	−0.50* (0.016)	−0.35 (0.098)	0.47* (0.026)	1.00

*^1^Global Severity Index T-score used for SCL-90-R. ^2^Total Stress T-score used for PSI-4-SF. ^3^CSI-32 raw score underwent cubic transformation to reduce negative skewness.^4^DCI raw score. ^5^ABC-2 Total raw score. ^6^SRS-2 Total T-score.*

^∼^p < 0.100, *p < 0.050, **p < 0.010, ***p < 0.001. The bold values indicates significant values.

**TABLE 12:** Pearson correlations for paternal variables (*n* = 23).

Variable	1.	2.	3.	4.	5.	6.
1. SCL-90-R^1^	1.00					
2. PSI-4-SF^2^	0.64** (0.001)	1.00				
3. CSI-32^3^	−0.25 (0.243)	−0.68*** (<0.001)	1.00			
4. DCI^4^	−0.40 (0.058)	−0.47* (0.022)	0.52* (0.011)	1.00		
5. ABC-2^5^	0.37 (0.082)	0.59** (0.003)	−0.46* **(0.028)**	−0.39 (0.063)	1.00	
6. SRS-2^6^	0.55** (0.007)	0.62** (0.001)	−0.54** **(0.008)**	−0.27 (0.216)	0.72*** **(<0.001)**	1.00

*^1^Global Severity Index T-score used for SCL-90-R. ^2^Total Stress T-score used for PSI-4-SF. ^3^CSI-32 raw score underwent cubic transformation to reduce negative skewness. ^4^DCI raw score. ^5^ABC-2 Total raw score. ^6^SRS-2 Total T-score.*

^∼^p < 0.100, *p < 0.050, **p < 0.010, ***p < 0.001. The bold values indicates significant values.

The significant correlations between the ABC-2 and SRS-2 scores for both mothers and fathers (see Tables 11, 12) indicated that they should not be included together in the MLMs for Aim 4. There was also a significant correlation between mothers’ SRS-2 scores and the Vineland-3 Adaptive Behavior Composite (*r* = −0.72, *p* = 0.001) and a marginally significant correlation between fathers’ SRS-2 scores and the Vineland-3 Adaptive Behavior Composite (*r* = −0.36, *p* = 0.085). Therefore, the ABC-2 Total Score, Vineland-3 Adaptive Behavior Composite, child age, and parent sex were included as predictors in the MLMs for Aim 4, as was the interaction between the ABC-2 Total Score and parent sex. Table 13 presents the results of the MLM analyses for mental health challenges, parenting stress, couples satisfaction, and dyadic coping.

**TABLE 13:** Multilevel model results for Aim 4 (*n* = 46).

	β (SE)			
	Mental health challenges	Parenting stress	Couples satisfaction	Dyadic coping
**Fixed Effects**				
Intercept	56.58*** (1.73)	55.91*** (1.16)	5.29*** (0.32)	128.51*** (2.68)
Challenging Behavior	0.23** (0.07)	0.21*** (0.05)	−0.03** (0.01)	−0.27** (0.10)
Adaptive Behavior	0.001 (0.15)	−0.21* (0.10)	0.07* (0.03)	0.21 (0.25)
Child Age	−0.50 (1.28)	−0.89 (0.86)	−0.34 (0.23)	0.55 (2.04)
Parent Sex	0.77 (1.72)	−0.51 (1.16)	−0.13 (0.18)	−3.22^∼^ (1.90)
Challenging Behavior × Parent Sex	−0.03 (0.06)	−0.02 (0.05)	−0.01 (0.01)	0.08 (0.08)
**Random Effects**				
Residual ($\sigma_{e}^{2}$)	134.34	61.60	1.42	163.09
Intercept ($\sigma_{u}^{2}\,0$)	0.89	1.29e-17	1.63	94.18
**Goodness-of-fit**				
AIC	367.19	335.75	207.95	389.43
BIC	381.82	350.38	222.58	404.06

^∼^p < 0.100, *p < 0.050, **p < 0.010, ***p < 0.001. The bold values indicates significant values.

#### Prediction of Parent Mental Health Challenges

As expected, there was a significant main effect of child challenging behaviors on parental mental health challenges (*p* = 0.001), such that higher levels of child challenging behaviors predicted elevated levels of mental health challenges. However, there were no significant main effects of child adaptive behavior, child age, or parent sex on parent mental health challenges, nor was there a significant interaction between child challenging behaviors and parent sex. Model diagnostics suggested that a linear regression model would be sufficient for predicting mental health challenges. The results of a linear regression model were similar to the results of the multilevel model.

#### Prediction of Parenting Stress

As expected, there was a significant main effect of child challenging behaviors on parenting stress (*p* < 0.001), such that higher levels of challenging behaviors predicted elevated parenting stress. There was also a significant main effect of child adaptive behavior on parenting stress, with higher levels of adaptive behavior predicting reduced parenting stress (*p* = 0.045). There were no significant main effects of child age or parent sex on parenting stress, nor was there a significant interaction between child challenging behaviors and parent sex. Model diagnostics suggested that a linear regression model would also be sufficient for predicting parenting stress. The results of a linear regression model were similar to the results of the multilevel model.

#### Prediction of Couples Satisfaction

As expected, there was a significant main effect of child challenging behaviors on couples satisfaction (*p* = 0.002), such that higher levels of child challenging behaviors predicted reduced couples satisfaction. There was also a significant main effect of child adaptive behavior on couples satisfaction (*p* = 0.015), with higher levels of adaptive behavior predicting greater couples satisfaction. There were no significant main effects of child age or parent sex on couples satisfaction, nor was there a significant interaction between child challenging behaviors and parent sex.

#### Prediction of Dyadic Coping

As expected, there was a significant main effect of child challenging behaviors on dyadic coping (*p* = 0.006), with higher levels of child challenging behaviors predicting poorer dyadic coping. There was also a marginally significant main effect of parent sex on dyadic coping (*p* = 0.091). In reference to the overall mean, fathers reported lower levels of dyadic coping compared to mothers. There were no significant main effects of child adaptive behavior or child age on dyadic coping, nor was there a significant interaction between child challenging behaviors and parent sex.

## Discussion

The current study was designed to examine aspects of the broader family environment in families of young children with FXS, including maternal and paternal well-being, features of the couples’ relationship, and relationships between child characteristics and parent and couple functioning. Findings suggest that both mothers and fathers of young children with FXS are at risk for experiencing significant mental health challenges and parenting stress. Unfortunately, nearly half of all mothers and fathers reported clinically significant levels of mental health challenges on the SCL-90-R. These rates are notably higher than expected given rates in general population for both males and females (64). An abundance of past research has established that women with the *FMR1* premutation experience mental health problems independent of the stress associated with parenting a child or multiple children with significant challenges (5). The results of the current study confirm that fathers in these families are also experiencing substantial mental health problems and that both parents may be in need of greater support and services.

Additionally, 43% of mothers and 30% of fathers reported clinically significant levels of parenting stress in the Difficult Child domain on the PSI-4-SF. Parents reported greater stress in the Difficult Child domain compared to the other domains (i.e., Parental Distress and Parent-Child Dysfunctional Interaction), suggesting that their perceptions of the child’s behavior were contributing more to their stress than their adjustment to parenting or their relationship with their child. This profile of parenting stress is consistent with past research on mothers of children with FXS (47, 65). Interestingly, there were no significant correlations between parents’ scores on the SCL-90-R or the PSI-4-SF. The lack of associations between parents’ scores on these measures potentially suggests that within families, one parent may be compensating for or supporting a partner who is struggling with mental health or parenting stress, potentially buffering against negative effects on the child. However, on the SCL-90-R, there was overlap for six families such that both the mother and father reported clinically significant levels of mental health challenges. Four of these mothers and two of these fathers reported previous psychiatric diagnoses, and in three of these families, there was another child with a disability. Additionally, two of these mothers and four of these fathers endorsed scores that fell in the severe range on the SRS-2, and four mothers and three fathers endorsed elevated levels of child challenging behavior on the ABC-2. Two mothers and four fathers endorsed scores in the severe range on the SRS-2, and four mothers and three fathers endorsed elevated levels of challenging behavior. Developing a better understanding of these dynamics within families is an important issue for future research. Another important consideration regarding these findings is that the majority of these data were collected during the COVID-19 pandemic, which may have contributed to parents’ mental health challenges and stress related to parenting, especially given that services for children with developmental disabilities were severely interrupted during this time (66–70). However, consistent with the results of the current study, past studies of parents of children with disabilities have also found that mothers and fathers experience elevated mental health challenges and stress [e.g., (30, 39–42, 48)].

Despite experiencing challenges with mental health and parenting stress, most mothers and fathers reported moderate to high levels of couples satisfaction and dyadic coping, with very few parents reporting notable relationship dissatisfaction, and a majority of parents reporting dyadic coping in the average or above average range. In these families, higher levels of couples satisfaction and dyadic coping may be protective against the daily stressors that the parents are experiencing (7). Importantly, features of the couples’ relationship are likely to affect the mother-child and father-child relationships. Specifically, parents with higher levels of couples satisfaction and dyadic coping may be more likely to engage in positive and responsive interactions with their children (8, 71–73). However, one limitation of these results is the possibility of selection bias such that only relatively satisfied couples were willing to participate in the current study.

Mothers and fathers also reported independently on their child’s challenging behaviors and ASD symptoms. Interspousal correlations indicated high degrees of correspondence between mothers’ and fathers’ scores on the SRS-2, but not the ABC-2. On average, mothers and fathers reported moderate levels of challenging behaviors that were similar to the ABC scores reported in Sansone et al. (60). With regard to ASD symptoms, a majority of both mothers and fathers reported scores that fell within the moderate to severe range, indicating that many of the children in this sample were demonstrating deficiencies in reciprocal social behavior that may interfere with everyday social interactions. Although significant correspondences were found between mothers’ and fathers’ scores on the SRS-2, mothers on average reported higher levels of autistic-like behaviors in the RRB subscale compared to fathers. Mothers may be more likely to observe these behaviors, especially given that 16 of the 23 families in the study reported that the mother spent more time with the child compared to the father. This difference in time spent with the child may also account for the lack of correspondences between mothers’ and fathers’ scores on the ABC-2. Fathers may be observing fewer of the child’s challenging behaviors when the mother is the child’s primary caregiver.

There were also some interesting differences in the correlations between maternal and paternal measures. For mothers (but not fathers), there were strong and significant correlations between the ABC-2 and the other measures of individual, couple, and child functioning (i.e., the SCL-90-R GSI score, the PSI-4-SF Total Stress T-Score, the CSI-32 raw score, the DCI raw score, and the SRS-2 Total T-score). However, for fathers (but not mothers), the SRS-2 was strongly correlated with every measure except the DCI. This finding may be due to differences in parental experiences of challenging behaviors and ASD symptoms; that is, mothers may be experiencing and managing more challenging behaviors compared to fathers, and fathers may be more concerned about or influenced by the child’s ASD symptoms compared to mothers. In particular, paternal parenting stress was associated with the child’s ASD symptoms, whereas maternal parenting stress was not. However, consistent with past research, parenting stress for both mothers and fathers was related to child challenging behaviors (7, 65). Future studies should investigate the similarities and differences between mothers and fathers further to determine how parents’ impressions of child behavior influence their well-being.

Additionally, parenting stress was found to associate with both couples satisfaction and dyadic coping, with no significant differences found between mothers and fathers. We also found a negative association between parent education and dyadic coping, which was unexpected, but should be explored in future studies. Parents with higher levels of education may experience more work-related stress that could negatively affect their individual well-being and their relationship with their partner. Additionally, child challenging behavior was also found to associate with parental mental health challenges, parenting stress, couples satisfaction, and dyadic coping. Surprisingly, no significant differences were found between mothers and fathers across these analyses, including any differences between mothers and fathers based on child challenging behaviors. Perhaps future investigations with larger and more diverse sample sizes would find differences between parents. Child adaptive behavior was also found to associate with couples satisfaction and parenting stress. These findings emphasize the importance of early intervention for children with FXS focused not only on communication and socialization skills, but also daily living skills that promote independence.

Interventions focused on reducing parenting stress in these families could also have a positive impact on parents’ individual well-being and the couples’ relationship. One potential intervention that could be beneficial for parents of children with FXS is Mindfulness-Based Stress Reduction (MBSR). MBSR is an established and empirically supported stress-reduction intervention that has been shown to reduce parental stress, depressive symptoms, and parent-reported child behavior problems in families of children with developmental disabilities (74, 75). Another study of MBSR for parents of children with developmental disabilities also found improvements in child social skills that were mediated by parent-child relational factors (i.e., attachment and discipline practices) (76). Interestingly, a recent study of mothers of children with FXS found that trait mindfulness, acceptance, and mindful parenting were associated with lower levels of anxiety, depression, and stress (77), providing additional evidence that mindfulness interventions may be beneficial for these families. Based on the findings of the current study, reductions in parental stress are likely to benefit parental individual well-being and couple well-being with anticipated benefits for the parent-child relationship as well.

Parent-implemented interventions focused on teaching parents strategies for managing child challenging behaviors and engaging in responsive interactions may also benefit parental and couple well-being in families of children with FXS. A recent study by Hall et al. (78) examined the effects of functional communication training (FCT) delivered *via* telehealth on problem behaviors in young boys with FXS. Children with FXS often engage in problem behaviors that serve different communicative functions, including gaining access to attention or a highly preferred item or escaping a demanding task or situation. The focus of FCT is to ensure that these problem behaviors are no longer reinforced by the caregiver while simultaneously teaching the child alternative and appropriate ways to communicate their preferences and needs. The FCT intervention conducted by Hall et al. (81) led to significant reductions in child problem behaviors as well as decreased levels of parenting stress, likely benefiting the entire family system.

Hall et al. and colleagues’ FCT study, along with other parent-implemented intervention studies conducted in the past several years with families of children with FXS (e.g., 78, 79), support the use of telehealth as an effective service delivery model for families of children with FXS. The use of telehealth-enabled interventions in this relatively rare population also allows families from rural and/or underserved communities to participate in research studies and receive services that may otherwise not be available to them (66, 80, 81). Telehealth also offers more flexibility compared to in-person services and is more cost-effective (66, 82). Therefore, telehealth methods for conducting assessments with and delivering interventions to families affected by FXS are likely to be utilized more frequently in the post-pandemic world.

### Limitations and Future Directions

There are some notable limitations to this study, including the relatively small sample size and the lack of diversity in the sample. FXS research studies focused on parent-child relationships tend to have small samples with a majority of the sample being two-parent households who identify as white, highly educated, and have household incomes in the middle to high range. Therefore, future studies should attempt to reduce barriers to participation in research for FXS families from underrepresented groups. These barriers include age of diagnosis, lack of information about research opportunities, time commitment for participation in research, and low household income (83, 84). Future research should identify ways to reduce these barriers and extend outreach to groups underrepresented in FXS research.

One notable strength of the study is the inclusion of both mothers and fathers as independent informants given that the majority of past research in FXS has focused on the mother-child dyad. Fathers have been historically underrepresented in research on child and adolescent development, both in the general population and in families that include children with disabilities, despite the fact that fathers have a unique and independent role in parenting compared to mothers and may differentially affect the child’s development (85–87). For decades, scholars have recognized the importance of the father’s role in the family and made suggestions for future research that involve conceptualizing the family as a complex and dynamic system (88–90), but very little progress has been made in this regard, particularly as it concerns families that include a child with a developmental disability (48, 91). Future studies in these families should continue to include fathers, and also consider differences between two-parent families, single-parent families, as well as between families of different-sex parents and same-sex parents. These approaches will increase understanding of the family as a complex and dynamic system that differentially influences the development of each family member.

Additionally, parents provided the measures of child ASD symptoms and challenging behaviors as opposed to these behaviors being rated by an independent informant. Future studies should incorporate multiple distinct assessments of both parent and child functioning to ensure accurate measurement within various domains of behavioral and psychological functioning given that self-report measures can be biased. Furthermore, biological markers of stress were not collected nor were any measures of IQ. Future studies could benefit from including these variables. Another limitation was the focus on concurrent associations as opposed to longitudinal ones. Future studies should examine relationships between parent and child functioning over time to develop a better understanding of how these relationships fluctuate as the child develops. Further, information was collected from families regarding the types of services and therapies being provided to the child/family. Nearly all families were receiving a combination of developmental services. Future studies should gather information regarding the quality of these services and the extent to which service provision affects parental and couple functioning. Finally, given that the majority of families participated in the study during the COVID-19 pandemic, the data reported in the current study may not reflect family functioning in families of children with FXS during more typical historical periods.

## Conclusion

The findings from the current study indicate the importance of considering the entire family system in families affected by FXS. Both mothers and fathers are in need of greater support to reduce their mental health challenges and parenting stress, which would likely benefit not only parental well-being, but also the couples’ relationship and the relationships between each parent and the child. The results of the current study also provide evidence that child challenging behaviors and limited adaptive functioning influence the couples’ relationship as well as individual parent functioning. Early intervention for children with FXS, parent-implemented interventions focused on managing challenging behaviors, and parent interventions focused on reducing stress are likely to benefit these families. Moreover, although many parents reported experiencing significant mental health challenges and parenting stress, this was not true for all mothers and fathers. Additionally, for many families, the couples’ relationship may be a source of strength that potentially buffers against some of the daily stressors faced by these families. Future studies should seek to identify protective factors in these families that support parent and family well-being and continue to investigate the complex dynamics between mothers, fathers, and children in families affected by FXS.

## Data Availability Statement

The raw data supporting the conclusions of this article will be made available by the authors, without undue reservation.

## Ethics Statement

The studies involving human participants were reviewed and approved by Institutional Review Board at the University of California, Davis. Written informed consent to participate in this study was provided by the participants’ legal guardians/next of kin.

## Author Contributions

The submitted research study was part of SP’s dissertation research project, which she completed under the guidance of LA. SP, AS, and LA were responsible for the initial conceptualization of the study. DH provided support with planning and conducting the analyses. SP wrote the first draft and made subsequent edits to the manuscript, based on feedback from DH, AS, and LA. All authors contributed to the interpretation of the data and approved the final manuscript.

## Conflict of Interest

The authors declare that the research was conducted in the absence of any commercial or financial relationships that could be construed as a potential conflict of interest.

## Publisher’s Note

All claims expressed in this article are solely those of the authors and do not necessarily represent those of their affiliated organizations, or those of the publisher, the editors and the reviewers. Any product that may be evaluated in this article, or claim that may be made by its manufacturer, is not guaranteed or endorsed by the publisher.
